# 2140. Biological Features of Response to VE303, a Defined Bacterial Consortium, in Patients with *Clostridioides difficile* Infection (CDI): Results from the Phase 2 CONSORTIUM Study

**DOI:** 10.1093/ofid/ofad500.1763

**Published:** 2023-11-27

**Authors:** Rajita Menon, Emily Crossette, Shakti Bhattarai, Vanni Bucci, Amanda L Prince, Jeremiah Faith, Bernat Olle, Jeffrey L Silber, Jason Norman

**Affiliations:** Vedanta BIosciences, Cambridge, Massachusetts; Vedanta Biosciences, Somerville, Massachusetts; UMASS Chan Medical School, 368 Plantation St, Massachusetts; UMass Chan Medical School, Worcester, Massachusetts; Vedanta Biosciences, Inc, Cambridge, Massachusetts; Icahn School of Medicine at Mount Sinai, New York, New York; Vedanta Biosciences, Somerville, Massachusetts; Vedanta Biosciences, Somerville, Massachusetts; Vedanta Biosciences, Somerville, Massachusetts

## Abstract

**Background:**

Fecal transplant and other donor-derived treatments promote a gut environment resistant to CDI, but these treatments have inherently variable quality attributes, are difficult to scale, and can transfer harmful pathogens. VE303 is a rationally defined consortium of 8 purified, clonal strains, overcoming the limitations of donor-derived treatments. In the CONSORTIUM Study, high-dose VE303 was well tolerated, reduced the odds of recurrent CDI (rCDI) by > 80%compared with placebo, and led to both robust colonization of VE303 strains and early restoration of the native microbiota. VE303 strain detection was associated with clinical efficacy.

**Figure:**
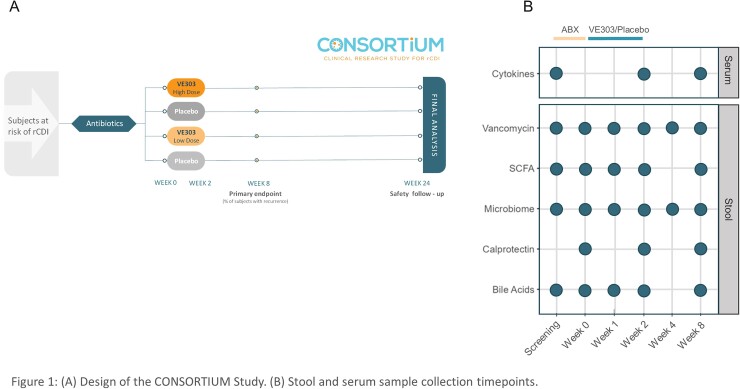

**Methods:**

This was a double-blind, placebo-controlled phase 2 trial (Fig. 1). After completion of antibiotic treatment for a lab-confirmed CDI episode, 79 subjects were randomized to low-dose VE303, high-dose VE303, or placebo and dosed orally once daily for 14 days. Subjects were followed for 24 weeks to monitor safety and rCDI episodes; samples were collected during dosing and at weeks 4 and 8 for metagenomic, metabolomic, and immune analysis. Random forest classification models were used to identify variables that predicted strain colonization and response to VE303.

**Results:**

On-study CDI recurrence in VE303-dosed subjects was predicted by higher primary bile acid (BA) levels, and lower levels of secondary BAs and short-chain fatty acids (SCFA) at screening. In particular, taurochenodeoxycholic acid predicted recurrence; lithocholic acid, deoxycholic acid, hexanoate and isovalerate predicted non-recurrence (Fig. 2). Advanced age was an important predictor of recurrence in all models (Fig. 2A, C). In models of VE303 colonization, particularly of the effector strain VE303-08 (Fig. 3A), low strain detection was predicted by high screening concentrations of primary BAs and inflammatory cytokines (Fig. 3B).

Metabolite profiles at screening predict response to VE303.

**Figure:**
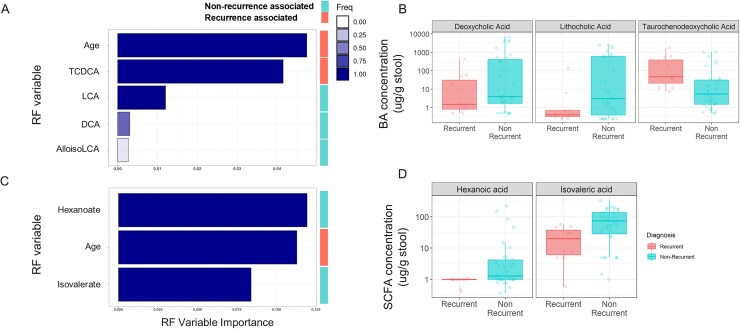

(A, C) Random Forest (RF) model importance plots show variables predicting on-study CDI recurrence in VE303-dosed subjects based on screening concentrations of BAs (top) and SCFAs (bottom). Variable importance is defined as the drop in predictive power when a variable is held out of the model. Bar color indicates the fraction of model iterations (out of 10) wherein the variable was found to be important. Vertical legend indicates association with recurrence. (B, D) Distributions of important BAs (top) and SCFAs (bottom) at screening in recurrent vs non-recurrent subjects. The box-and-whisker plots depict the median, interquartile range (IQR, at the top and bottom of the boxes), and reasonable extreme values at 1.5 X IQR in the dataset (where the vertical lines end).

Low prevalence of effector strain VE303-08 is predicted by inflammation at screening.

**Figure:**
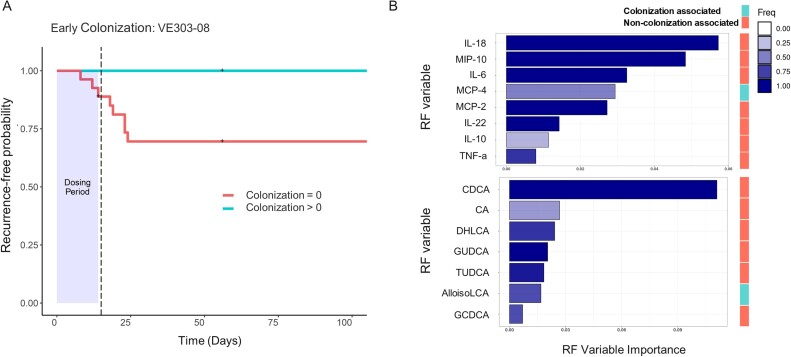

(A) In subjects dosed with VE303, detection of VE303-08 at the end of dosing was associated with a lower CDI recurrence rate and greater recurrence-free probability (adjusted p = 0.08, log rank test). (B) RF model importance plots show variables predicting VE303-08 detection during Day 1 to Day 28 based on screening concentrations of serum cytokines (top) and BAs (bottom). Variable importance is defined as the drop in predictive power when a variable is held out of the model. Bar color indicates the fraction of model iterations (out of 10) wherein the variable was found to be important.

**Conclusion:**

In subjects at high risk of rCDI, BA concentrations at screening (high primary BAs, low secondary BAs) were predictive of on-study CDI recurrence. Furthermore, high screening levels of primary BAs and inflammatory cytokines predicted low VE303 colonization. Taken together, this suggests that inflammation and metabolite levels may be important considerations when treating patients with rCDI.

**Disclosures:**

**Rajita Menon, PhD**, Vedanta Biosciences: Patent inventor|Vedanta Biosciences: I am employed by Vedanta Biosciences.|Vedanta Biosciences: Stocks/Bonds **Emily Crossette, PhD**, Vedanta Biosciences: Patent Inventor|Vedanta Biosciences: Employee, Stock options **Amanda L. Prince, PhD**, Vedanta Biosciences, Inc: Inventor|Vedanta Biosciences, Inc: Employee|Vedanta Biosciences, Inc: Stocks/Bonds **Jeremiah Faith, PhD**, Vedanta Biosciences: Advisor/Consultant **Bernat Olle, PhD**, Vedanta Biosciences, Inc.: Board Member|Vedanta Biosciences, Inc.: Employee, receive salary and equity compensation|Vedanta Biosciences, Inc.: Ownership Interest **Jeffrey L. Silber, MD**, Vedanta Biosciences: Employee, Stock Options **Jason Norman, PhD**, Vedanta Biosciences: Patent Inventor|Vedanta Biosciences: Employee, Stock Options

